# Return to sport after surgical treatment for high-grade (Rockwood III–VI) acromioclavicular dislocation

**DOI:** 10.1007/s00167-019-05528-w

**Published:** 2019-05-14

**Authors:** D. E. Verstift, C. L. Welsink, A. J. Spaans, M. P. J. van den Bekerom

**Affiliations:** Department of Orthopaedic Surgery, OLVG, Postbus 95500, 1090 HM Amsterdam, The Netherlands

**Keywords:** Acromioclavicular dislocation, AC joint, Rockwood, Return to sport, Functional outcome, Systematic review

## Abstract

**Purpose:**

Acromioclavicular (AC) joint dislocations are common in a young and active population, especially in people performing contact sports. Full recovery with a fast and high rate of return to sport is desirable. This systematic review aims to combine patient outcomes in order to help surgeons in addressing patient expectations regarding the return to sport after surgical intervention for AC dislocations.

**Methods:**

To conduct this systematic review, the PRISMA guidelines were followed. Articles were included if written in English or Dutch and evaluated return to sport after any type of surgical intervention for Rockwood types III to VI AC dislocations in patients practicing sports. Outcome parameters were return to sport, time to return to sport, level of sport, functional outcome scores and complications.

**Results:**

Twelve studies involving 498 patients were included, of which 462 patients practiced sports. 432 (94%) patients returned to sport. The weighted mean time to return to sport was 4.0 months. 338 out of 401 patients (84%) returned to the same level of pre-injury sport and 35 patients (9%) lowered their level of sport. The weighted mean Constant score was 92 out of 100.

**Conclusion:**

The rate of return to sport after surgical intervention for Rockwood (RW) III–VI AC dislocations is high. However, the level of evidence was low and due to the methodological heterogeneity between studies, subgroup analyses of return to sport outcomes were not feasible.

**Level of evidence:**

Systematic review of level I–IV studies, level IV.

## Introduction

Acromioclavicular (AC) joint injuries are common in young athletes with an incidence of 9.2 per 1000 person-years [27]. The incidence of AC injuries is higher in contact sports and is highest in adults in their 20s [21, 31]. Full recovery with return to pre-injury level of sports and minimal time lost to injury is essential in the treatment of these injuries.

The Rockwood classification is most commonly used for classifying AC joint injuries according to the severity [31]. Most type I and II dislocations are treated conservatively. For type IV to VI AC joint dislocations, surgical treatment generally is the intervention of choice [21, 29, 31]. The optimal management of type III AC joint dislocations remains controversial. A trend is set toward initial non-surgical treatment, unless non-surgical treatment fails in patients with persistent pain or the inability to return to sport or work [21, 29, 31].

Many surgical techniques have been described for the repair of AC joint dislocations [2, 21]. When evaluating surgical treatment, existing literature mainly measures functionality with outcome scores or self-reported outcome measures. Limited literature exists about the rate of return to sport in this population. However, return to sport has been found to be a good indicator of success after treatment for injuries to the musculoskeletal body [10, 14, 17, 22]. Analyzing the rate of return to sport could provide surgeons and patients with relevant outcome information to optimize shared decision making. The purpose of this systematic review is to assess the rate of return to sport after surgical intervention in high-grade AC dislocation (Rockwood III–VI), to help surgeons address patient expectations. In contrast to the existing literature on this topic, the present review aims to combine outcomes of sports-practicing patients of studies with larger sample sizes and more recent studies with higher levels of evidence.

## Materials and methods

To conduct this systematic review, the PRISMA guidelines (Preferred Reporting Items for Systematic Reviews and Meta-Analyses) were followed [23].

### Search strategy

Using the search terms “acromioclavicular joint” and “return to sport/to play”, an electronic search was performed in MEDLINE (PubMed), EMBASE and Cochrane with the assistance of a clinical librarian. There was no restriction on the date of publication. The final search was conducted in September 2018. Based on the titles and abstracts, two reviewers (D.V. and C.W.) independently identified potentially relevant articles for review of the full text. For each identified article, the reference list was screened and a manual search was conducted for articles that could potentially be relevant.

### Inclusion and exclusion criteria

The included articles should be written in English or Dutch and evaluate the return to sport after any type of surgical intervention for high-grade (Rockwood III–VI) AC dislocation in patients practicing sports. There was no restriction on the type of sport, level of sport and age of the patient. Exclusion criteria were reviews of the literature, expert opinions, non-clinical studies, case reports and studies with a sample size of less than 20 athletes. Studies were also excluded when there were insufficient data on the number of patients practicing sports pre- and post-injury. Studies that also included non-athletes in the analysis were only included in this review when separate and sufficient data on the sports-practicing patients were presented.

### Data extraction

Data from eligible studies were extracted based on a predefined data extraction form. The following data and baseline parameters were recorded when available: author, publication year, study design, level of evidence, numbers of patients, sex, age, laterality, Rockwood classification (or Tossy III when the Tossy classification was used), acute or chronic injury (with definition of the interval according to the author), operation technique, type of sports, and follow-up time.

The primary objective was to determine the rate of return to sport. Secondary outcomes were the time to return to sport, level of sport pre- and post-injury, clinical outcome scores and complications. Self-reported outcome measures or clinical outcome scores were only considered for analysis when three or more studies reported on this outcome.

### Methodological quality

The methodological quality of included studies was assessed by assigning levels of evidence as previously defined by the Centre for Evidence-Based Medicine (http://www.cebm.net). Additionally, the quality of the included studies was evaluated according to the Methodological Index for Nonrandomized Studies (MINORS) checklist [33]. This instrument is designed to assess the methodological quality of non-randomized surgical studies. To assess risk of bias for randomized trials, the Cochrane Collaboration tool will be used. Bias is assessed as a judgment (high, low or unclear) for individual elements from five domains (selection, performance, attrition, reporting and other) [8]. A level of evidence was assigned by two authors (D.V. and C.W.). Disagreement was resolved by consensus.

### Statistical analysis

For the rate of return to sport, a forest plot was created (Graphpad Prism; Graphpad Software, San Diego, CA, USA) with confidence intervals calculated by a binominal exact calculation for proportions. *I*^2^ tests (MedCalc; Medcalc Software, Ostend, Belgium) were used to determine heterogeneity between studies. Values of *I*^2^ between 25 and 49% were considered low, 50–74% was considered moderate, and values greater than 75% were considered to have high statistical heterogeneity [9]. Further analysis on the outcomes was presented narratively. Means (weighted), medians, ranges and percentages were calculated using Microsoft Excel 2016 (Microsoft, Seattle, WA, USA).

## Results

**Fig. 1:**
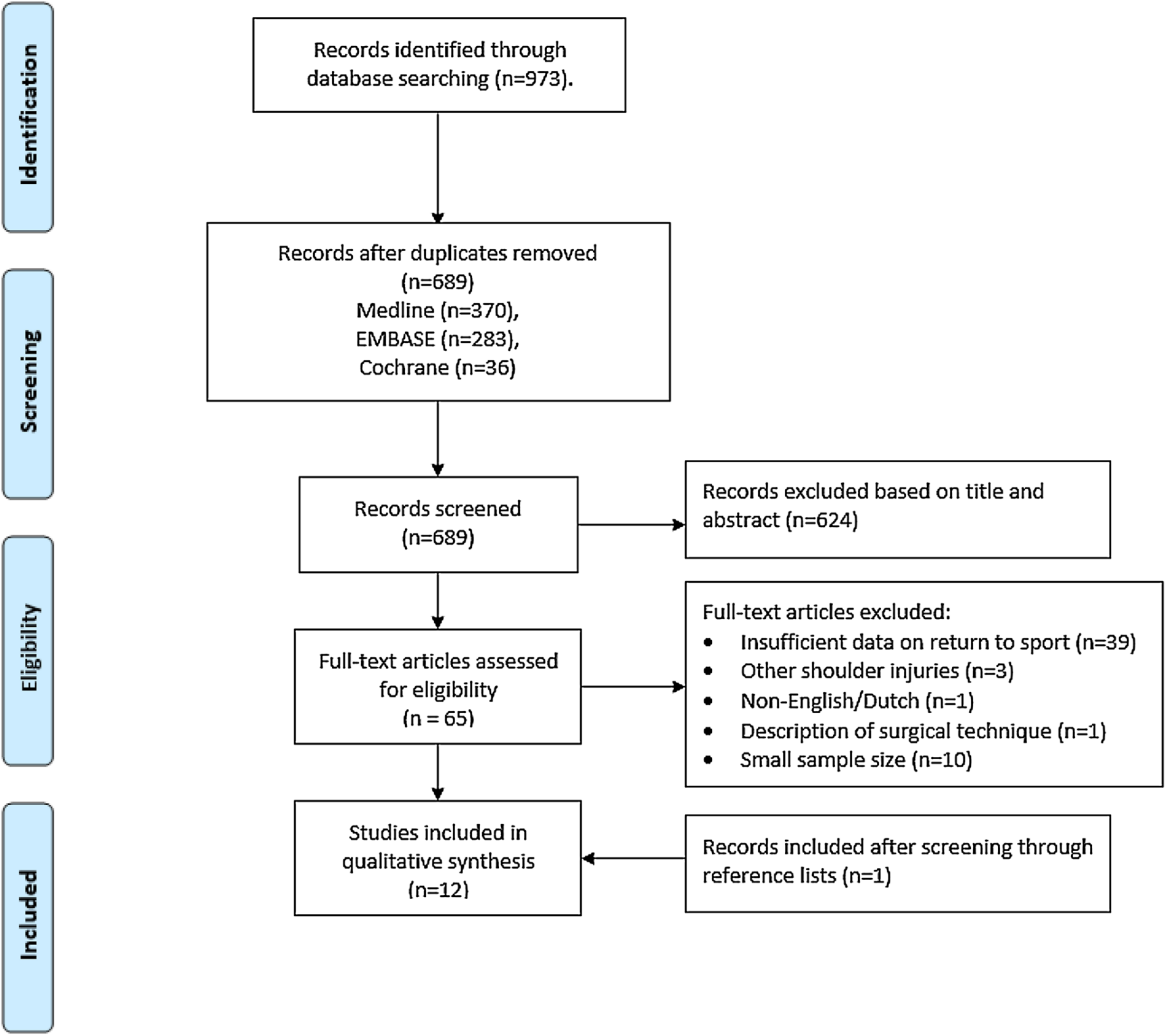
PRISMA flow diagram—inclusion of records

After removal of duplicates, our initial search yielded 689 articles (370 from PubMed/MEDLINE, 283 from Embase, 36 from Cochrane) (see Fig. 1). Sixty-five articles were selected for full review after screening through titles and abstracts. Eleven articles met the inclusion criteria and were suitable for data extraction. By screening through the reference lists, one additional article was found to be suitable for inclusion in the review [3]. Of the 12 included studies, there was 1 randomized controlled trial (RCT) (level I), 1 prospective comparative study (level II), 4 retrospective comparative studies (level IV) and 6 retrospective case series (level IV). The median MINORS score for the comparative studies was 16.5/24 (range 16–23) and the median MINORS score for the non-comparative studies was 10/16 (range 8–10). Areas of improvement for most studies according to the MINORS were prospective data collection, unbiased assessment of the study endpoint and the prospective calculation of the study size. The RCT was not blinded for patients, personnel and outcome assessment, thereby at risk for detection bias and performance bias. The loss to follow-up was 18%, increasing the risk of attrition bias. Due to the high variety of the different types of sports, level of sports and the surgical techniques, in addition to the methodological heterogeneity and retrospective nature of most study designs, data were not pooled in an official meta-analysis.

**Fig. 2:**
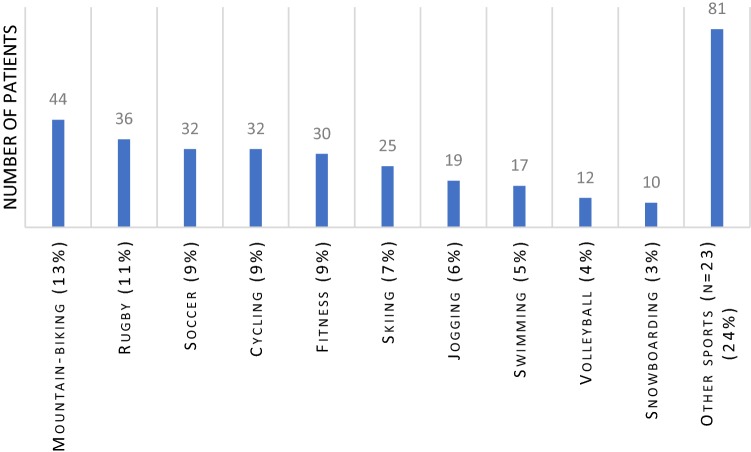
The distribution in type of sport participation

Among all included studies, 498 patients underwent surgery for AC dislocation. Baseline characteristics could not be stratified for athletes since many studies included mixed populations. There were 97 patients diagnosed with Tossy III, 93 patients with Rockwood III, 100 patients with Rockwood IV, 208 with Rockwood V and none with Rockwood VI. The weighted mean age was 34 years (range 28–46) with 87% males. The weighted mean follow-up was 34 months (range 22–48). Of all patients, 462 were athletes practicing all levels of sports, ranging from recreational to professional sports. The five most common sports practiced were mountain biking (13%), rugby (10%), soccer (9%), cycling (9%) and fitness (9%) (see Fig. 2).

Most studies used techniques focused on the fixation of the coracoclavicular (CC) ligaments (see Table 1). Older studies most commonly performed an open modified Weaver–Dunn technique or Kirschner wires with additional CC fixation. In more recent studies, open techniques with cortical buttons or AC- and CC-fixation with an allo- or autograft were most commonly used. Three studies performed an arthroscopic technique, all using double cortical button techniques. Due to the wide variety of different surgical techniques, an additional analysis on the return to sport for each technique was not feasible.

**Table 1:** Study characteristics

Author	Year	Level of evidence	MINORs	Patients; *n*	Male; *n*	Mean age; y (range)	Dominant side; *n*	Mean follow-up; months (range)	Acute/chronic (cut-off, weeks)	Type of surgery
Krueger-Franke [15]	1993	IV	10/16	21	20	40 (23–73)	NR	22	NR	Modified Kirschner wire with CC repair using PDS-cord augmentation
Weinstein [38]	1995	IV	16/24	44	37	32 (17–57)	34	48 (24–108)	Acute + chronic (3)	Modified Weaver–Dunn with CC repair using non-absorbable sutures
Tienen [35]	2003	IV	10/16	21	NR	33 (20–49)	NR	36 (4–55)	Acute	Modified Weaver–Dunn with AC repair using absorbable braided suture
Logters [18]	2008	IV	8/16	32	26	39 (25–63)	NR	36 (9–84)	Acute	Kirschner wire with CC repair using absorbable cord
De Carli [3]	2015	IV	16/24	30	30	29	NR	42 (24–96)	Acute (3)	CC repair using cortical buttons
Loriaut [19]	2015	IV	10/16	39	26	36 (20–55)	31	42 (24–60)	Acute (3)	CC repair using cortical buttons
Marcheggiani Muccioli [20]	2016	II	23/24	43	43	30 (19–54)	28	28	Chronic (6)	CC repair using LARS
Saier [32]	2016	IV	9/16	42	NR	35 (18–45)	NR	31 (24–61)	Acute (4)	CC repair using double cortical buttons (arthroscopic)
Garofalo [5]	2017	IV	10/16	32	24	28 (22–51) M	NR	30 (24–33)	Chronic	AC + CC repair using semitendinosus hamstring autograft
Porschke [30]	2017	IV	16/24	55	43	Non-overhead 46 M, overhead 33 (18–65)	NR	24 (18–45)	Acute (4)	CC repair using cortical buttons
Muller [24]	2018	I	NR	G1: 29 G2: 32	G1: 28 G2: 28	G1: 37.8 G2: 34.6	G1: 17 G2: 15	G1: 39.3 ± 22.7 G2: 30.8 ± 8.4	Acute (2)	G1: clavicular hookplate G2: double cortical buttons (arthroscopic)
Xu [39]	2018	IV	17/24	78	58	Single: 29 Double: 31	NR	NR	Acute (2)	Single vs double cortical buttons (arthroscopic)

*w* weeks, *mo* months, *y* years, *NR* not reported, *CC* coracoclavicular, *AC* acromioclavicular, *G1* Group 1, *G2* Group 2, *M* median data

For the postoperative care, all studies started with a period of immobilization, ranging from 2 to 6 weeks, followed by either physical therapy or exercises focusing on passive and active range of motion (ROM) (see Table 2).

**Table 2:** Return to sport outcomes

Author	Rockwood subtype	Rehabilitation protocol	Athletes; *n*	Return to sport; *n*	Return to sport protocol	Mean time to return to sport, w (range)	Level of Sports	Mean functional outcome measures
Krueger-Franke [15]	Tossy III	6 w immobilization → PT	21	19	NR	NR	Same level as pre-injury (90%)	NR
Weinstein [38]	Tossy III	4–6 w immobilization → PT	28	26	No contact sports < 36 w	NR	Same level as pre-injury (93%)	NR
Tienen [35]	V	4 w immobilization → after 6 w aROM	21	21	NR	10 (4–16)	Same level as pre-injury (86%), Lower level (14%)	Constant: 97 (66–100)
Logters [18]	Tossy III	2 w immobilization and 6 w of p/aROM	28	28	NR	NR	Same level as pre-injury (100%)	Not stratified for athletes
De Carli [3]	III	4 w immobilization, after 2 w pROM → after 8 w aROM	30	30	Contact sports and heavy work allowed > 12 w	17	Same level as pre-injury (83%), Lower level (17%)	Constant: 98
Loriaut [19]	III, IV	6 w immobilization and pROM → aROM	35	34	Avoidance of lifting, carrying, pushing and pulling < 12w	21	Same level as pre-injury (86%), Lower level (12%)	Not stratified for athletes
Marcheggiani Muccioli [20]	III, IV, V	3 w immobilization and pROM → aROM	43	43	Contact sport allowed > 8–12 w	Professionals: 16 (12–20) M Non-professionals: 20 (16–24)	Same level as pre-injury (100%)	Constant: prof: 97; non-prof: 91
Saier [32]	V	6 w immobilization and PT → fROM	42	42	Sport allowed > 12 w, overhead or contact sports allowed > 24 w	NR	Same level as pre-injury (62%), Lower level (38%)	Constant: 94 (86–100)
Garofalo [5]	V	6 w immobilization and pROM after 3 w → after 7 w aROM	32	30	Full activity including contact sports allowed after 16 w	NR	Same level as pre-injury (94%)	ASES: 85 (82–98)
Porschke [30]	V	4 w immobilization and pROM → after 6 w aROM	43	41	Non-contact sport allowed after 3 mo, no restrictions > 24 w	38 (12–72) M	Same level as pre-injury (79%), Lower level (16%)	Constant: non-overhead: 94 (49–100), overhead: 89 (63–100) M
Muller [24]	III, IV, V	6 w immobilization → aROM	61	59	Fitness sport allowed > 16 w, contact sports allowed > 40 w	NR	G1: SSAS 6.2 ± 1.5, ASOSS 77.8 ± 20.8 G2: SSAS 7 ± 1.4, ASOSS 91.4 ± 10.3	NR
Xu [39]	IV	6 w immobilization and pROM → aROM	78	59	Limited rehabilitation sports allowed > 12 w	Single button: 21 (12–32) Double button: 13 (12–16)	Same level as pre-injury (76%)	Constant: Single: 83 ± 4, Double: 92 ± 3

*w* weeks, *mo* months, *NR* not reported, *PT* physical therapy, *aROM* active range of motion, *pROM* passive range of motion, *fROM* free range of motion, *G1* Group 1, *G2* Group 2, *SSAS* shoulder sport activity score, *ASOSS* athletic shoulder outcome scoring system, *M* median data

### Return to sport

**Fig. 3:**
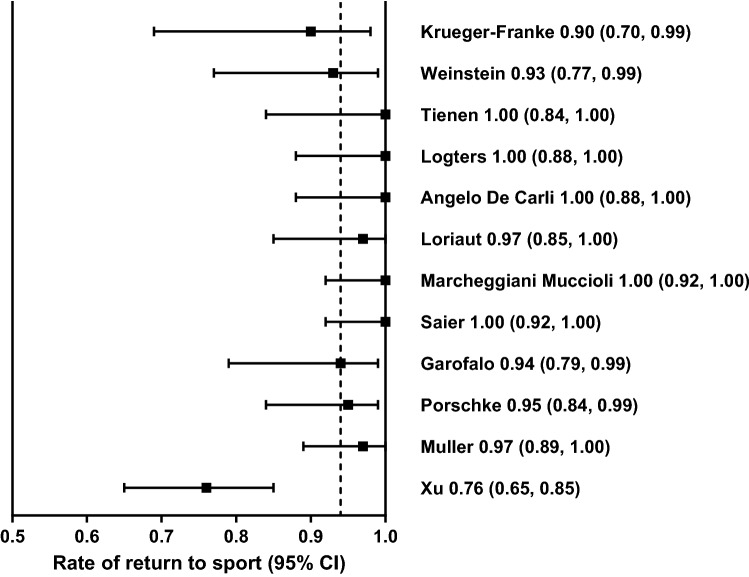
Forest plot of the rate of return to sport after surgical treatment for high-grade AC dislocation

A total of 462 patients practicing sports underwent surgery for high-grade AC dislocation. Of those, 432 patients (94%) returned to sport (see Table 2). Among the included studies, the mean rate of return to sport ranged from 76 to 100% (*I*^2^ = 74%, 49–84%) (see Fig. 3). In most studies, after physical therapy or ROM exercises, patients were allowed to return to sport after 3 months. In four studies, the mean time to return to sport was reported, ranging from 2.5 to 5.3 months [3, 19, 35, 39]. The weighted mean time to return to sports in these studies is 4.0 months. Eleven studies reported on the level of sport. Out of the 401 patients practicing sports in these studies, 338 (84%) returned to the same level of pre-injury sports, ranging from 62 to 100% (*I*^2^ = 80%, 63–88%) (see Table 2). 35 patients (9%) returned to sports at a lower level, ranging from 0 to 38% (*I*^2^ = 86%, 76–91%). The types of sports varied greatly among the studies; therefore, a subgroup analysis of the rate of return to sport for type of sport was not feasible.

### Acute or chronic

Among the 12 included studies, a variety of cut-off intervals for acute versus chronic surgery were used, ranging from 2 to 6 weeks (see Table 1). In four studies, the interval was not reported. Due to the heterogeneity of the definition for acute and chronic surgery, it was not possible to perform a subgroup analysis on return to sport outcomes for acute versus chronic surgery.

### Rockwood subtypes

For subgroup analysis of the rate of return to sport for the Rockwood subtypes, studies could only be included when stratified data for each subgroup were presented. Two studies provided sufficient data on the rate of return to sport for Rockwood type III injuries [3, 20]. The rate of return to sport was 100% among 53 patients. In two studies [20, 39], for Rockwood type IV, 64 out of 83 patients (77%) returned to sport. For Rockwood type V, five studies reported the rate of return to sport [5, 20, 30, 32, 35]. Among 153 patients, 149 (97%) returned to sport, ranging from 94 to 100% (*I*^2^ = 7%, 0–66%). No data on return to sport were provided for patients diagnosed with Rockwood type VI.

### Functional outcome scores

Many different functional outcome scores were used in the studies. Only the Constant score was used in more than three studies, in which the data on patients practicing sports were stratified. In six studies, the mean Constant score was reported [3, 20, 30, 32, 35, 39] (see Table 2). The weighted mean Constant score at follow-up for these studies was 92 out of 100 (range 84–98), indicating an excellent outcome. The mean time to follow-up in these studies ranged from 2 to 3.5 years.

### Complications and re-surgery

The data on complications could not be stratified for athletes alone. The following complication rates are combined for all included studies. Among 498 operated patients, the rate of complications was 5.2% at final follow-up. There were 6 cases (1.2%) of failure or migration of osteosynthesis material, 5 cases (1.0%) of complete loss of reduction, 10 cases (2.0%) of wound infection, 2 cases of tunnel misplacement, 1 case of coracoid fracture due to non-compliance and 2 subacromial bursitis. The rate of revision surgery due to complications in these studies was 3.0%.

## Discussion

The most important finding of the present study was a very high (94%) overall rate of return to sport in patients surgically treated for Rockwood III–VI AC dislocation. Additionally, many patients (84%) returned to their pre-injury level of sports. The rate of return to sport in patients surgically treated for high-grade AC dislocation is superior to that of patients surgically treated for rotator cuff repair and arthroscopic Bankart repair (81–85%) [14, 22].

Although this outcome is promising, it must be noted that 10 of 12 studies are level IV, of which the majority are non-comparative studies. In this review, only one randomized study could be included. The RCT of Muller et al. [24] showed that, at 24 months, 100% of patients treated with arthroscopically assisted double-suture-buttons participated in sports compared to 93% of patients treated with a clavicular hook plate.

A subgroup analysis on the rate of return to sport for different types of sports was not feasible. The study of Porschke et al. [30] compared sports activity for overhead athletes versus non-overhead athletes. Overhead athletes needed to change sport activity significantly more often (54% vs 12%; *p* = 0.011) and 27% had to reduce their sports level to low-demanding sports, while none of the non-overhead athletes lowered their level (*p* = 0.029). Muller et al. [24] found that overhead athletes had more benefit from a double-suture-button technique than collision athletes and suggested non-anatomic clavicle hookplate as a good alternative for the collision athletes.

The rates of return to sport for Rockwood III and V dislocations were similar. However, there were no comparative studies determining the return to sport outcomes for the different Rockwood subtypes. In addition, not all patients could be included in the Rockwood subgroup analysis since some authors used the Tossy classification, in which Tossy type III corresponds to Rockwood types III to VI. Notable is that the rate of return to sport in Rockwood IV dislocations was found to be lower, although this is mainly based on one study [39] analyzing 78 out of 83 patients with Rockwood IV dislocation included in this analysis. This study found a lower rate of return to former sports, especially among patients treated by a single cortical button technique. However, no data was presented on the possibility that patients practiced other sports at follow-up.

The surgical techniques for AC dislocation varied greatly among the included studies. A subgroup analysis was not feasible. A recent review of Gowd et al. [7] showed, although there is a trend toward minimally invasive procedures, there are no differences in loss of reduction, complication rate and revision rate between open and arthroscopic AC joint reconstruction. Considering return to sport outcomes, the RCT by Muller et al. [24] showed that patients treated arthroscopically by double cortical buttons had a superior level of performance in shoulder sport, close to the level of performance in healthy athletes, compared to patients treated by a hookplate.

Recently, a review on the return to sport after surgical management for AC dislocation was published by Kay et al. [13]. Five out of 12 studies included in the present review were also reviewed by Kay et al.; however, the present review included 7 additional studies reporting on return to sport outcomes after high-grade AC dislocation, one of which was an RCT. In contrast to Kay et al., we excluded three studies [4, 11, 37] due to lack of data on the practicing of sports pre-injury and four studies due to a sample size of less than 20 athletes [1, 6, 28, 36]. Another methodological difference was the approach to non-stratified data on mixed populations. The present review only combined patient outcomes from different studies when data on athletes were stratified, for instance in the analysis of functional outcome measures, while Kay et al. combined patient outcomes from all studies reporting on these outcomes, including studies with mixed populations. In addition, Kay et al. combined return to sport outcomes of acute versus chronic surgery although the cut-off intervals between studies were very different. No conclusion could be drawn on the rate of return to sport for acute versus chronic AC dislocation because of the many different definitions used for these injuries. For future research, it is important to work with one definition and one cut-off interval to be able to compare acute and chronic surgeries in different studies.

The present review was limited by the overall low level of evidence provided by the many level IV studies. As a consequence, no robust conclusions can be drawn on return to sport outcomes for different subgroups comparing surgical techniques, acute versus chronic surgery, types of sports and Rockwood subtypes. In addition, the rates of return to sport were mostly secondary outcomes in these studies, with insufficient attention paid to the many aspects of the return to sport. This may lead to publication bias when only high rates of return to sport are reported.

In this review, no comparison was made between conservative and surgical treatment for patients with high-grade AC dislocation. Non-operative treatment for high-grade AC dislocation is being studied more extensively since functional outcome may be non-inferior to surgery. A recent meta-analysis comparing surgical and conservative treatments of Rockwood III AC dislocation showed no significant differences in terms of functional outcome scores [34]. Similar findings are presented for Rockwood IV and V AC dislocations [12, 16, 25, 26]. Murray et al. [25], comparing open reduction and tunneled suspensory device with non-operative treatment in Rockwood III and V, states that non-operatively managed patient generally recover faster, although a substantial part of remain dissatisfied and require delayed surgical reconstruction.

The outcomes of this review may help surgeons in addressing patient expectations in a sporting population facing surgery for AC dislocation. Patients can now be properly informed on the rate of return to sport, time to return to sport and the patient satisfaction with the results. Still, surgeons need to consider conservative therapy, especially in Rockwood III, since non-surgical treatment can result in similar functional outcome. For future research, high-quality comparative studies are needed to provide adequate data for subgroup analysis on functional and sport outcomes, for both conservative and surgical treatment. When assessing return to sport outcomes, it is imperative to incorporate aspects of sports activity, frequency and intensity.

## Conclusion

Overall, the rate of return to sport following surgery for AC joint dislocation is high and most patients can expect to return to their pre-injury level of sport.

## Abbreviations

AC: Acromioclavicular
ASOSS: Athletic shoulder outcome scoring system
CC: Coracoclavicular
MINORS: Methodological Index for Nonrandomized Studies
NR: Not reported
PT: Physical therapy
RCT: Randomized controlled trial
ROM: Range of motion (aROM: active, pROM: passive, fROM: free)
RW: Rockwood
SSAS: Shoulder sport activity score
VAS: Visual analog score
